# Supplementary material for: Identification and Pathogenicity of Fusarium Fungi Associated with Dry Rot of Potato Tubers
Source: Microorganisms. 2024 Mar 16;12(3):598. doi: 10.3390/microorganisms12030598 (PMC10975030; doi:10.3390/microorganisms12030598)
Supplement: Supplementary file 1 [file microorganisms-12-00598-s001.zip › microorganisms-2861458-supplementary.pdf]

Table S1. *Fusarium* strains whose sequences were used in the phylogenetic study.

Species	Species complex	Strain ID	Substrate	Origin	GenBank accessions	
					tef	*rpb2*
F. nelsonii	FCSC	NRRL 13338			MW233225	MW233569
F. acaciae-mearnsii	FSAMSC	NRRL 26755	Australian acacia	South Africa	MW233086	MW233429
F. aethiopicum	FSAMSC	NRRL 46726	Wheat seed	Ethiopia	MW233126	MW233470
F. armeniacum	FSAMSC	FRC R-6472	Slash pine seedling	USA	MW233156	MW233500
F. armeniacum	FSAMSC	FRC R-8519	Corn soil	USA	MW233191	MW233535
F. asiaticum	FSAMSC	NRRL 13818	Barley	Japan	MW233069	MW233412
F. austroamericanum	FSAMSC	NRRL 28585	Herbaceous vine	Brazil	MW233095	MW233438
F. boothii	FSAMSC	NRRL 26916	Corn	South Africa	MW233088	MW233431
F. brachygibbosum	FSAMSC	NRRL 20954	Sorghum	India	MW233075	MW233418
F. brachygibbosum	FSAMSC	FRC R-8851	Pearl millet	Niger	MW233198	MW233542
F. brasilicum	FSAMSC	NRRL 31238	Barley	Brazil	MW233104	MW233448
F. cerealis	FSAMSC	NRRL 13721	Potato	Poland	MW233068	MW233411
F. cortaderiae	FSAMSC	NRRL 29297	Toetoe (*Cortaderia* sp.)	New Zealand	MW233098	MW233442
F. culmorum	FSAMSC	NRRL 25475	Barley kernel	Denmark	MW233082	MW233425
F. dactylidis	FSAMSC	NRRL 29380		USA	MW233099	MW233443
F. gerlachii	FSAMSC	NRRL 36905	Wheat	USA	MW233118	MW233462
F. goolgardii	FSAMSC	KOD 1087	Grass tree (*Xanthorrhoea glauca*)	Australia	MW233218	MW233562
F. goolgardii	FSAMSC	KOD 1090	Grass tree (*Xanthorrhoea glauca*)	Australia	MW233219	MW233563
F. graminearum	FSAMSC	NRRL 31084	Corn	USA	MW233103	MW233447
F. kyushuense	FSAMSC	NRRL 3509	Wheat seed	Japan	MW233056	MW233399
F. kyushuense	FSAMSC	NRRL 6491	Vinyl plate	Japan	MW233057	MW233400
F. langsethiae	FSAMSC	NRRL 34176			MW233111	MW233455
F. langsethiae	FSAMSC	NRRL 36236			MW233114	MW233458
F. longipes-4	FSAMSC	NRRL 20695	Soil	USA	MW233073	MW233416
F. longipes-4	FSAMSC	NRRL 39691			MW233123	MW233467
F. louisianense	FSAMSC	NRRL 54197		USA	MW233134	MW233478
F. lunulosporum	FSAMSC	NRRL 13393	Grapefruit	South Africa	MW233063	MW233406
F. meridionale	FSAMSC	NRRL 28436	Sweet potato	New Caledonia	MW233092	MW233435
F. mesoamericanum	FSAMSC	NRRL 25797	Banana	Honduras	MW233083	MW233426
F. musarum	FSAMSC	NRRL 28507	Banana	Panama	MW233094	MW233437
F. nepalense	FSAMSC	NRRL 54222		Nepal	MW233135	MW233479

Species	Species complex	Strain ID	Substrate	Origin	GenBank accessions	
					tef	*rpb2*
F. nodosum	FSAMSC	NRRL 13431	Tomato stem	Turkey	MW233065	MW233408
F. nodosum	FSAMSC	NRRL 36351	Stored peanuts	Portugal	MW233117	MW233461
F. palustre	FSAMSC	NRRL 43289	Spartina rhizosphere soil		MW233124	MW233468
F. palustre	FSAMSC	NRRL 54056	Smooth cordgrass *(Spartina alterniflora*)	USA	MW233131	MW233475
F. pentaseptatum	FSAMSC	FRC R-9121	Soil	China	MW233213	MW233557
F. pentaseptatum	FSAMSC	NRRL 66939	Soybean root	Anda, China	MW233217	MW233561
F. pentaseptatum	FSAMSC	LLC 1022	endophytic from seed of *Striga hermonthica*	Ethiopia	OP487255	OP486819
F. poae	FSAMSC	NRRL 31280	Oat (var. Linhagem 469)	Brazil	MW233105	MW233449
F. poae	FSAMSC	NRRL 36285	Barley	Norway	MW233115	MW233459
F. praegraminearum	FSAMSC	NRRL 39664	Litter in corn field	New Zealand	MW233121	MW233465
F. pseudograminearum	FSAMSC	NRRL 28062	Barley crown	Australia	MW233090	MW233433
F. robustum	FSAMSC	NRRL 13392	Paraná pine (*Araucaria angustifolia*)	Argentina	MW233062	MW233405
F. sambucinum	FSAMSC	NRRL 13394		Germany	MW233064	MW233407
F. sambucinum	FSAMSC	NRRL 20663		Germany	MW233071	MW233414
F. sambucinum	FSAMSC	NRRL 20666	Potato	United Kingdom	MW233072	MW233415
F. sambucinum	FSAMSC	NRRL 31964	Scotch broom (*Cytisus scoparius*)	New Zealand	MW233106	MW233450
F. sambucinum	FSAMSC	NRRL 31969	Gorse (*Ulex europaeus*)	New Zealand	MW233107	MW233451
F. sambucinum	FSAMSC	FRC R-738	Potato	USA	MW233142	MW233486
F. sambucinum	FSAMSC	FRC R-4712	Potato tuber	Switzerland	MW233148	MW233492
F. sibiricum	FSAMSC	NRRL 53429	Oat	Russia	MW233129	MW233473
F. sibiricum	FSAMSC	NRRL 53430	Oat	Russia	MW233130	MW233474
F. sp. nov.-6	FSAMSC	NRRL 28066	Corn	Japan	MW233091	MW233434
F. sp. nov.-6	FSAMSC	NRRL 54640	Sweet potato stem	Papua New Guinea	MW233136	MW233480
F. sp. nov.-6	FSAMSC	NRRL 54683	Sweet potato stem	Papua New Guinea	MW233137	MW233481
F. sp. nov.-7	FSAMSC	NRRL 46743	Wheat seed	Ethiopia	MW233127	MW233471
F. sp. nov.-7	FSAMSC	NRRL 66736		Ethiopia	MW233139	MW233483
F. sp. nov.-8	FSAMSC	NRRL 29296	Chayote (*Sechium edule*)	New Zealand	MW233097	MW233441
F. sp. nov.-9	FSAMSC	NRRL 13465	Tiger pear (*Opuntia aurantica*)	Argentina	MW233067	MW233410
F. sp. nov.-9	FSAMSC	NRRL 66925	Tiger pear (*Opuntia aurantica*)	Argentina	MW233162	MW233506
F. sp. nov.-10	FSAMSC	NRRL 22189	Soybean	Brazil	MW233076	MW233419
F. sp. nov.-10	FSAMSC	FRC R-7403	Potato	South Africa	MW233166	MW233510

Species	Species complex	Strain ID	Substrate	Origin	GenBank accessions	
					tef	*rpb2*
F. sp. nov.-11	FSAMSC	NRRL 22192	Palm tree	Indonesia	MW233077	MW233420
F. sp. nov.-12	FSAMSC	FRC R-642	Carnation 'Iroquois'		MW233141	MW233485
F. sp. nov.-12	FSAMSC	FRC R-7107	Soil	South Africa	MW233163	MW233507
F. sp. nov.-12	FSAMSC	FRC R-7122	Soil	South Africa	MW233164	MW233508
F. sp. nov.-12	FSAMSC	FRC R-8227	Debris	South Africa	MW233188	MW233532
F. sp. nov.-12	FSAMSC	NRRL 66929	Debris	South Africa	MW233189	MW233533
F. sp. nov.-13	FSAMSC	NRRL 39635	Toetoe (*Cortaderia* sp.)	New Zealand	MW233120	MW233464
F. sp. nov.-13	FSAMSC	NRRL 39685	Toetoe (*Cortaderia* sp.)	New Zealand	MW233122	MW233466
F. sp. nov.-14	FSAMSC	NRRL 36134			MW233113	MW233457
F. sp. nov.-15	FSAMSC	NRRL 66921	Wheat straw	South Africa	MW233146	MW233490
F. sp. nov.-15	FSAMSC	FRC R-8154	Soil	South Africa	MW233185	MW233529
F. sp. nov.-15	FSAMSC	FRC R-8203	Debris	South Africa	MW233187	MW233531
F. sp. nov.-16	FSAMSC	NRRL 66927	Natural vegetation soil	South Africa	MW233178	MW233522
F. sp. nov.-16	FSAMSC	FRC R-8136	Debris	South Africa	MW233184	MW233528
F. sp. nov.-17	FSAMSC	NRRL 26795	Soil	USA	MW233087	MW233430
F. sp. nov.-27	FSAMSC	FRC R-8881		Nigeria	MW233201	MW233545
F. sporotrichioides	FSAMSC	NRRL 3299	Corn	France	MW233055	MW233398
F. sporotrichioides	FSAMSC	NRRL 29131	Oat kernel	Germany	MW233096	MW233440
F. subflagellisporum	FSAMSC	COAD 2989	Branches of *Mangifera indica* trees	Brazil	MT774486	MZ970426
F. subtropicale	FSAMSC	NRRL 66764	Barley	Paraná, Brazil	MW233140	MW233484
F. transvaalense	FSAMSC	FRC R-6827	Natural vegetation debris	South Africa	MW233157	MW233501
F. transvaalense	FSAMSC	FRC R-6855	Natural vegetation debris	South Africa	MW233158	MW233502
F. ussurianum	FSAMSC	NRRL 45681	Oat	Russia	MW233125	MW233469
F. venenatum	FSAMSC	NRRL 22196	Corn	Germany	MW233078	MW233421
F. venenatum	FSAMSC	NRRL 25413		England	MW233080	MW233423
F. venenatum	FSAMSC	NRRL 26228	Halmbase of winter wheat	Austria	MW233085	MW233428
F. venenatum	FSAMSC	NRRL 32015	Soil	Australia	MW233109	MW233453
F. vorosii	FSAMSC	NRRL 37605		Hungary	MW233119	MW233463
F. acutisporum	FSSC	NRRL 22574 T	*Coffea arabica*	Guatemala	LR583593	LR583814
F. addoense	FSSC	CBS 146510 T	*Citrus sinensis*, crown	South Africa	MW248741	MW446575
F. addoense	FSSC	CBS 146508	*Citrus sinensis*, crown	South Africa	MW248739	MW446573
F. akasia	FSSC	PPRI 27978 T	*Euwallacea perbrevis* specimen	Indonesia	MT009951	MT009931

Species	Species complex	Strain ID	Substrate	Origin	GenBank accessions	
					tef	*rpb2*
F. akasia	FSSC	PPRI 27980	*Euwallacea perbrevis* specimen	Indonesia	MT009954	MT009937
F. ambrosium	FSSC	NRRL 22346 T	Gallery of *Euwallacea fornicatus* in *Camellia sinensis*	India	FJ240350	EU329503
F. ambrosium	FSSC	NRRL 20438	*Euwallacea fornicatus* insect body in the gallery formed in tea tree (*Camellia sinensis*)	India	AF178332	JX171584
F. amplum	FSSC	BBA 4170 T	*Coffea* sp.	East Africa	LR583594	LR583815
F. awan	FSSC	PPRI 27973T	*Euwallacea similis* specimen	Indonesia	MT009973	MT009919
F. awan	FSSC	PPRI 27975	*Euwallacea similis* specimen	Indonesia	MT009974	MT009922
F. bataticola	FSSC	NRRL 22400	*Ipomoea batatas*	USA	AF178343	EU329509
F. bataticola	FSSC	NRRL 22402 T	*Ipomoea batatas*	USA	AF178344	FJ240381
F. borneense	FSSC	NRRL 22579 T	Bark of a recently dead unidentified tree	Indonesia	AF178352	EU329515
F. bostrycoides	FSSC	NRRL 36253 T	Soil	Honduras	LR583597	LR583818
F. bostrycoides	FSSC	CBS 102824	Leaf litter	Colombia	LR583596	LR583817
F. breve	FSSC	MUCL 16108 T	Soilwater	Belgium	LR583601	LR583822
F. breve	FSSC	CPC 27190	*Citrus sinensis*, dry root rot	Italy	LT746199	LT746312
F. breviconum	FSSC	NRRL 22659T	*Gladiolus* sp.	Indonesia	LR583600	LR583821
F. catenatum	FSSC	UTHSC 09-1008	Zebra shark (*Stegostoma fasciatum*)	USA	KC808213	KC808354
F. catenatum	FSSC	UTHSC 09-1009 T	Zebra shark (*Stegostoma fasciatum*)	USA	KC808214	KC808355
F. crassum	FSSC	MUCL 11420 T		France	LR583604	LR583823
F. crassum	FSSC	NRRL 46703	Nematode egg	Spain	HM347126	EU329661
F. cryptoseptatum	FSSC	NRRL 22412 T	Bark	French Guiana	AF178351	EU329510
F. cucurbiticola	FSSC	NRRL 22399 T	*Cucurbita ficifolia*	Netherlands	DQ247592	LR583825
F. cucurbiticola	FSSC	NRRL 22153	Cucurbit	USA	AF178346	EU329492
F. cyanescens	FSSC	CBS 518.82 T	Human foot	Netherlands	LR583605	LR583826
F. cyanescens	FSSC	CBS 637.82	Human foot	Netherlands	LR583606	LR583827
F. diminutum	FSSC	MUCL 18798 T	treated wood of *Coelocaryon preussii*		LR583607	LR583828
F. diminutum	FSSC	LC 13825	Acer palmatum	Japan	MW620164	MW474689
F. drepaniforme	FSSC	NRRL 62941	Unknown woody host	Singapore	KM406626	KM406647
F. duplospermum	FSSC	NRRL 62583 T	Oral mycangium of an ambrosia beetle (*Euwallacea perbrevis*) trapped in an avocado (*Persea americana*) grove	USA	KC691553	KC691642

Species	Species complex	Strain ID	Substrate	Origin	GenBank accessions	
					tef	*rpb2*
F. duplospermum	FSSC	NRRL 62589	Oral mycangium of an ambrosia beetle (*Euwallacea perbrevis*) trapped in an avocado (*Persea americana*) grove	USA	KC691552	KC691641
F. epipeda	FSSC	CPC 38310 T	*Bouvardia* sp. imported from Uganda	Netherlands	MW834285	MW834022
F. epipeda	FSSC	CPC 38311	*Bouvardia* sp. imported from Uganda	Netherlands	MW834286	MW834023
F. euwallaceae	FSSC	NRRL 54722T	Live ambrosia beetle (*Euwallacea* sp. IS/CA) infecting avocado tree (*Persea americana* cv. Hass) grown in an orchard	Israel	JQ038007	JQ038028
F. euwallaceae	FSSC	NRRL 62626	*Euwallacea* sp. on *Acer negundo*	USA	KC691532	KU171702
F. falciforme	FSSC	IMI 268681 T	Human mycetoma	USA	LT906669	LT960558
F. falciforme	FSSC	CBS 121450	Declined grape vine	Syria	JX435161	JX435261
F. falciforme	FSSC	LC 11569	*Vitis* sp.	China	MW620165	MW474690
F. ferrugineum	FSSC	NRRL 32437 T	Human subcutaneous nodule	Switzerland	DQ246979	EU329581
F. ferrugineum	FSSC	CPC 28194	*Citrus sinensis*	Italy	LR583602	LT746341
F. floridanum	FSSC	NRRL 62628 T	Mycangium of *Euwallacea interjectus* infesting *Acer negundo*	USA	KC691535	KC691624
F. floridanum	FSSC	NRRL 62606	Gallery of *Euwallacea interjectus* infesting *Acer negundo*	USA	KC691533	KC691622
F. gamtoosensis	FSSC	CPC 37120 T	*Citrus sinensis*, crown	South Africa	MW248762	MW446611
F. gannanense	FSSC	NJFU-JX12 T	Oral mycetangium of a *Euwallacea interjectus* beetle infesting a trunk of a live *Triadica cochinchinensis*	China	LC701609	LC701981
F. gannanense	FSSC	NJFU-JX01	*Euwallacea interjectus* beetle infesting in trunks of dying *Alniphyllum fortunei*	China	LC701625	LC701973
F. haematococcum	FSSC	G.J.S. 02-90 T	Dying tree	Sri Lanka	DQ247510	LT960561
F. helgardnirenbergiae	FSSC	NRRL 22387 T	Bark of unidentified tree	French Guiana	AF178339	EU329505
F. hypothenemi	FSSC	NRRL 52782 T	*Hypothenemus hampei*, adult	Benin	JF740850	JF741176
F. hypothenemi	FSSC	NRRL 52783	*Hypothenemus hampei*, adult	Uganda	JF740851	JF741177
F. illudens	FSSC	NRRL 22090 T	Beilschmiedia tawa	New Zealand	AF178326	JX171601
F. kelerajum	FSSC	FRC S-1836	On branch of recently dead unidentified tree	Sri Lanka	DQ247515	LR583835
F. kelerajum	FSSC	G.J.S. 02-122 T	Trunk of fallen, live tree	Sri Lanka	DQ247518	

Species	Species complex	Strain ID	Substrate	Origin	GenBank accessions	
					tef	*rpb2*
F. keratoplasticum	FSSC	FRC S-2477 T	Indoor plumbing	USA	JN235712	JN235897
F. keratoplasticum	FSSC	MUCL 18301	Greenhouse humic soil	Belgium	LR583613	LR583836
F. kuroshium	FSSC	UCR3641 T	Gallery wall produced by *Euwallaceae* sp. in *Platanus racemosa* (California sycamore)	USA	KX262216	LR583837
F. kuroshium	FSSC	NRRL 62946	*Euwallacea* sp. on *Platanus racemosa*		KM406630	KM406650
F. kurunegalense	FSSC	CBS 119599 T	Recently cut non-native tree standing in private garden	Sri Lanka	DQ247511	LR583838
F. lerouxii	FSSC	CBS 146514 T	*Citrus sinensis*, root scaffold	South Africa	MW248768	MW446617
F. lichenicola	FSSC	CBS 623.92 T	Human foot	Germany	LR583620	LR583845
F. liriodendri	FSSC	NRRL 22389 T	*Liriodendron tulipifera*	USA	AF178340	EU329506
F. macrosporum	FSSC	CPC 28191 T	*Citrus sinensis*, crown	Italy	LT746218	LT746331
F. macrosporum	FSSC	CPC 28193	*Citrus sinensis*, crown	Italy	LT746220	LT746333
F. mahasenii	FSSC	FRC S-1845 T	Small dead branch on living tree	Sri Lanka	DQ247513	LT960563
F. mahasenii	FSSC	FRC S-1840	Rotting wood	Sri Lanka	DQ247520	
F. martii	FSSC	CBS 115659	*Solanum tuberosum* var. Maritta	Germany	JX435156	JX435256
F. martii	FSSC	CBS 142423	*Citrus sinensis*, crown	Italy	LT746216	LT746329
F. mekan	FSSC	PPRI 27971 T	*Euwallacea similis* specimen	Indonesia	MT009964	MT009916
F. mekan	FSSC	PPRI 27972	*Euwallacea similis* specimen	Indonesia	MT009965	MT009917
F. merkxianum	FSSC	CPC 38701 T	*Chrysanthemum* sp. imported from Uganda	Netherlands	MW834288	MW834025
F. merkxianum	FSSC	CPC 38702	*Chrysanthemum* sp.	Netherlands	MW834289	MW834026
F. metavorans	FSSC	CBS 135789 T	Human pleural effusion, lung cancer	Greece	LR583627	LR583849
F. metavorans	FSSC	NRRL 22654	*Malus sylvestris*	Italy	DQ247636	LR583848
F. mori	FSSC	NRRL 22230 T	*Morus alba*, twig	Japan	AF178358	EU329499
F. neerlandicum	FSSC	CBS 232.34 T	*Pisum sativum*	Netherlands	MW847906	MW847903
F. neocosmosporiellum	FSSC	NRRL 22166 T	*Heterodera glycines*	USA	AF178350	EU329497
F. neocosmosporiellum	FSSC	NRRL 22468	*Arachis hypogaea*, stored nut	Guinea-Bissau	KM231933	LR583901
F. ngaiotongaense	FSSC	CBS 126407 T	Tree bark	New Zealand	LR583621	LR583846
F. ngaiotongaense	FSSC	LC 13833	*Armeniaca mume*	Japan	MW620175	MW474700
F. noneumartii	FSSC	FRC S-0661 T	*Solanum tuberosum*	Israel	LR583630	LR583852
F. noneumartii	FSSC	Fs112	*Solanum lycopersicum*	USA	DQ164848	

Species	Species complex	Strain ID	Substrate	Origin	GenBank accessions	
					tef	*rpb2*
F. noneumartii	FSSC	Fs306	*Solanum lycopersicum*	USA	DQ164847	
F. obliquiseptatum	FSSC	NRRL 62611 T	Gallery of an ambrosia beetle (*Euwallacea* sp. 3) infesting an avocado tree (*Persea americana*)	Australia	KC691548	KC691637
F. obliquiseptatum	FSSC	NRRL 62610	*Euwallacea* sp. on *Persea americana*	Australia	KC691547	KC691636
F. oblongum	FSSC	NRRL 28008 T	Human eye	USA	LR583631	LR583853
F. oblongum	FSSC	LC 7499	Carbonatite	China	MW620179	MW474704
F. oligoseptatum	FSSC	NRRL 62579 T	Live female *Euwallacea validus* beetle, extracted from a gallery in a *Ailanthus altissima* tree	USA	KC691538	LR583854
F. oligoseptatum	FSSC	NRRL 62578	*Euwallacea validus* on *Ailanthus altissima*	USA	KC691537	KC691626
F. papillatum	FSSC	NRRL 62944	Mycangium of a living female TSHB beetle (*Euwallacea perbrevis*) from a gallery in a branch of an infested tea (*Camellia sinensis*)	Sri Lanka	KM406627	KM406648
F. papillatum	FSSC	NRRL 62943 T	Mycangium of a living female TSHB beetle (*Euwallacea perbrevis*) from a gallery in a branch of an infested tea (*Camellia sinensis*)	Sri Lanka	KM406628	
F. paraeumartii	FSSC	NRRL 13997 T	*Solanum tuberosum*, decaying stem base	Argentina	DQ247549	LR583855
F. paraeumartii	FSSC	LC13836	*Castanopsis fargesii*	China	MW620181	MW474706
F. parceramosum	FSSC	CBS 115695 T	Soil	South Africa	JX435149	JX435249
F. parceramosum	FSSC	NRRL 31158	Human wound	USA	DQ246916	EU329559
F. paulenelsonii	FSSC	CBS 309.75 T	*Pisum sativum*		MW847907	MW847904
F. perseae	FSSC	CPC 26829 T	*Persea americana*, trunk canker lesions	Italy	LT991902	LT991909
F. perseae	FSSC	CPC 26831	*Persea americana*, trunk canker lesions	Italy	LT991904	LT991911
F. petroliphilum	FSSC	NRRL 13952	*Pelargonium* sp. root	South Africa	DQ246835	LR583857
F. petroliphilum	FSSC	CBS 398.66	*Saccharum officinarum*	Brazil	LR583633	LR583859
F. phaseoli	FSSC	CBS 265.50	*Phaseolus* sp.	USA	HE647964	KM232375
F. phaseoli	FSSC	NRRL 22743	*Glycine max*	Brazil	AY320145	EU329525
F. piperis	FSSC	NRRL 22570 T	*Piper nigrum*	Brazil	AF178360	EU329513
F. plagianthi	FSSC	NRRL 22632	*Hoheria glabrata*	New Zealand	AF178354	JX171614

Species	Species complex	Strain ID	Substrate	Origin	GenBank accessions	
					tef	*rpb2*
F. populicola	FSSC	NJFU-DF02 T	Oral mycetangium of *Euwallacea interjectus* beetle infesting the trunk of a living poplar	China	LC701596	LC701960
F. populicola	FSSC	NJFU-JS03	Oral mycetangium of *Euwallacea interjectus* beetle infesting the trunk of a living poplar	China	LC701602	LC701966
F. protoensiforme	FSSC	NRRL 22178 T	Dicot tree, bark	Venezuela	AF178334	EU329498
F. pseudensiforme	FSSC	NRRL 46517 T	Recently dead tree, bark	Sri Lanka	DQ247512	KC691645
F. pseudensiforme	FSSC	NRRL 22653	*Cocos nucifera*, leaf	Indonesia	DQ247635	LR583868
F. pseudopisi	FSSC	CBS 266.50 T	*Pisum sativum*		MW834290	MW834027
F. pseudoradicicola	FSSC	NRRL 25137 T	Diseased cocoa pods	Papua New Guinea	JF740757	JF741084
F. pseudotonkinense	FSSC	CBS 143038 T	Human, scrape of cornea	Netherlands	LR583640	LR583867
F. quercinum	FSSC	NRRL 22652 T	Quercus cerris wood, declined tree	Italy	DQ247634	LR583869
F. quercinum	FSSC	NRRL 22611	Human cornea	USA	DQ246841	EU329518
F. rectiphorus	FSSC	G.J.S. 9296 T	Recently dead tree	Sri Lanka	DQ247509	LR583871
F. rectiphorus	FSSC	NRRL 22396	Bark	French Guiana	AF178342	EU329508
F. regulare	FSSC	CBS 230.34 T	*Pisum sativum*	Netherlands	LR583643	LR583873
F. regulare	FSSC	CBS 190.35	*Phaseolus* sp.	USA	LR583642	LR583872
F. rekanum	FSSC	PPRI 27163 T	*Acacia crassicarpa* infested with *Euwallacea perbrevis* (TSHBa) beetle	Indonesia	MN249151	MN249137
F. rekanum	FSSC	CMW 51760	*Acacia crassicarpa* infested with *Euwallacea perbrevis*	Indonesia	MN249160	MN249146
F. riograndense	FSSC	URM 7361 T	Human nasal cavity	Brazil	KX534002	KX534003
F. samuelsii	FSSC	CBS 114067 T	Bark	Guyana	LR583644	LR583874
F. silvicola	FSSC	CBS 123846 T	*Liriodendron tulipifera*, fallen trunk	USA	LR583646	LR583876
F. solani	FSSC	NRRL 66304 T	Tuber of *Solanum tuberosum* (potato) collected from field following harvest	Slovenia	KT313611	KT313623
F. solani	FSSC	CBS 101018	Raspberry	Italy	LR583651	LR583878
F. solani	FSSC	LC 13849	Soil	China	MW620196	MW474721
F. solani-melongenae	FSSC	CBS 225.58	Unsterilized dewaxed cotton duck	Panama	LR583609	LR583830
F. solani-melongenae	FSSC	NRRL 22657	*Capsicum annuum*	Netherlands	DQ247639	LR583831
F. spathulatum	FSSC	UTHSC 98-1305 T	Human synovial fluid	USA	DQ246882	EU329542

Species	Species complex	Strain ID	Substrate	Origin	GenBank accessions	
					tef	*rpb2*
F. stercicola	FSSC	CBS 142480			KY556525	KY556553
F. stercicola	FSSC	KU 90.15 –			MG237868	MG237866
F. stercicola	FSSC	KU 90.1.15 –			MG237869	MG237867
F. stercicola	FSSC	CBS 142481 T	Compost yard waste plant debris	Germany	LR583658	LR583887
F. suttonianum	FSSC	NRRL 32858 T	Human wound	USA	DQ247163	EU329630
F. suttonianum	FSSC	CBS 124892	Human nail	Gabon	JX435139	JX435239
F. tonkinense	FSSC	IMI 113868 T	*Musa sapientum*	Vietnam	LT906672	LT960564
F. tonkinense	FSSC	CBS 118931	*Solanum lycopersicum*	UK	LR583662	LR583891
F. tuaranense	FSSC	NRRL 22231 T	Pará rubber tree (*Hevea brasiliensis*) damaged by an unknown ambrosia beetle	Malaysia	KC691542	KC691631
F. tuaranense	FSSC	NRRL 46518	*Hevea brasiliensis*	Malaysia	KC691543	KC691632
F. tumidispermum	FSSC	NJFU-JX26 T	*Euwallacea interjectus* beetle infesting the trunk of a living *Elaeocarpus decipiens*	China	LC701621	LC701993
F. vanettenii	FSSC	NRRL 45880 T	Progeny from the sexual cross of parents from *Pisum sativum* and soil from a potato field	USA	LR583636	LR583862
F. vanettenii	FSSC	CBS 127118	Soil	USA	LR583637	LR583863
F. variasi	FSSC	PPRI 27958 T	*Acacia crassicarpa* infested with *Euwallacea perbrevis* (TSHBa)	Indonesia	MT009967	MT009913
F. variasi	FSSC	PPRI 27968	*Acacia crassicarpa* infested with *Euwallacea perbrevis* (TSHBa)	Indonesia	MT009969	MT009915
F. venezuelense	FSSC	NRRL 22395 T	Bark	Venezuela	AF178341	EU329507
F. waltergamsii	FSSC	NRRL 32323 T	Human bronchoalveolar lavage fluid	USA	DQ246951	EU329576
F. waltergamsii	FSSC	NRRL 22655	Three-plywood	Nigeria	DQ247637	LT960559
F. warna	FSSC	PPRI 27974 T	*Euwallacea perbrevis* (TSHBa) specimen	Indonesia	MT009955	MT009920
F. warna	FSSC	PPRI 27977	*Euwallacea perbrevis* (TSHBa) specimen	Indonesia	MT009958	MT009925
F. yamamotoi	FSSC	NRRL 22277 T	*Zanthoxylum piperitum*, trunk	Japan	AF178336	FJ240380
Neocosmospora anhuiensis	FSSC	CGMCC 3.24869 T	rotten twigs	China	OQ866530	OQ866525
N. aquatica	FSSC	CGMCC 3.24275 T	submerged decaying wood in a stream	China	OQ064518	
N. aquatica	FSSC	CGMCC 3.24276	submerged decaying wood in a stream	China	OQ064520	
N. aurantia	FSSC	CGMCC 3.24866 T	rotten bark	China	OQ866528	OQ866523

Species	Species complex	Strain ID	Substrate	Origin	GenBank accessions	
					tef	*rpb2*
N. caricae	FSSC	CBS 148865 T	Trunk of *Ficus carica* cv. Sabz	Iran	OK539518	OK415859
N. caricae	FSSC	CBS 148932	Trunk of *Ficus carica* cv. Sabz	Iran	OK539516	OK415857
N. dimorpha	FSSC	CGMCC 3.24867 T	rotten twigs	China	OQ866529	OQ866524
N. galbana	FSSC	CGMCC 3.24868 T	rotten bark	China	OQ866532	OQ866527
N. geoasparagicola	FSSC	CPC 40592 T	Field soil cultured with *Asparagus officinalis*	Netherlands	ON745622	ON759301
N. geoasparagicola	FSSC	CPC 40628	Field soil cultured with *Asparagus officinalis*		ON745627	ON759306
N. lechatii	FSSC	CPC 42648 T	Unidentified dead wood	French Guiana	OP481884	OP481882
N. lechatii	FSSC	CPC 42649	Unidentified dead wood	French Guiana	OP481885	OP481883
N. lithocarpi	FSSC	LC 1113 T	*Lithocarpus glaber* (also called *Lithocarpus glabra*)	China	MW620172	MW474697
N. lithocarpi	FSSC	LC 13831	*Lithocarpus glabra*	China	MW620173	MW474698
N. magnoliae	FSSC	MFLUCC 17-2615 T	dried fruits of *Magnolia champaca*	Thailand	MT212207	MT212200
N. maoershanica	FSSC	CGMCC 3.24870 T	twigs	China	OQ866531	OQ866526
Geejayessia atrofusca		CBS 125482	*Staphylea trifolia*, twigs	Canada	MW834282	HQ897775
